# Self-Rated Healthy Life Expectancy Changes in Jiangxi Province of China by Gender and Urban–Rural Differences, 2013–2018

**DOI:** 10.3389/fpubh.2020.596249

**Published:** 2021-01-25

**Authors:** Zhitao Liu, Huilie Zheng, Yuhang Wu, Shengwei Wang, Yong Liu, Songbo Hu

**Affiliations:** Jiangxi Province Key Laboratory of Preventive Medicine, School of Public Health, Nanchang University, Nanchang, China

**Keywords:** expansion of morbidity, healthy life expectancy, life expectancy, self-rated health, urban-rural

## Abstract

**Background:** Globalization has brought about rapid economic and technological development, and life expectancy (LE) is constantly increasing. However, it is not clear whether an increase in LE will result in an increase in healthy life expectancy (HLE). This study evaluates trends in the self-rated healthy life expectancy (SRHLE) of residents aged 15 and older in Jiangxi Province of China from 2013 to 2018 and analyzes gender differences and urban–rural differences. This study provides a basis for the formulation of relevant public health policies.

**Methods:** Based on two National Health Services Survey databases of Jiangxi in 2013 and 2018 as well as infant mortality rates and under-5 mortality rates from the Health Commission of Jiangxi, the Sullivan method was used to calculate SRHLE. The changes in SRHLE were decomposed into health and mortality effects using the decomposition method.

**Results:** SRHLE decreased from 56.55 to 55.54 years and from 60.00 to 57.87 years for men and women aged 15 from 2013 to 2018, respectively. The SRHLE of women aged 15 was 3.45 and 2.34 years longer than that of men in 2013 and 2018, respectively. The SRHLE of urban men aged 15 was 2.9 and 4.46 years longer than that of rural men in 2013 and 2018, respectively, and that of urban women aged 15 was 3.28 and 5.57 years longer than that of rural women.

**Conclusions:** The decreased SRHLE indicated that the self-rated health (SRH) status of residents in Jiangxi has worsened, and it provided evidence for the expansion of morbidity, mainly due to the increased prevalence of chronic diseases and the improvement in residents' health awareness. Policy efforts are necessary to control the increased morbidity of chronic diseases and reduce gender and urban–rural differences in the quantity and quality of years lived.

## Introduction

With the development of the social economy, previously high rates of human mortality decrease to low levels, and the health status of the population continues to improve. The decline in mortality means a continuous increase in life expectancy (LE). However, it remains unclear whether the increased LE is accompanied by an increase in healthy life expectancy (HLE). The results of a global burden of disease (GBD) study showed that in most countries, the increase in HLE was smaller than the increase in overall LE from 1990 to 2017, indicating more years lived in poor health (1).

Population health assessment is essential for health care planning at the national and global levels (2). In the past few decades, global LE has continued to increase, and child mortality has decreased significantly (3). It is in this context that people begin to pay attention to the relationship between longevity and health, and the notion of healthy expectancy emerged to address this issue (4). This article explores the changes in LE and health levels of male–female urban–rural residents in Jiangxi (China) from 2013 to 2018 and to reveal the trend of differences in LE and health levels between men and women and between urban and rural residents.

Healthy expectancy is generated based on LE. Sanders first proposed the concept prototype “productive man-years” in 1964 (5). In 1971, Sullivan first used disability-free LE in his report. It used the life table principle to deduct the survival time in the disabled state to obtain LE without disability (6). Healthy expectancy refers to the number of years that a certain age group is expected to survive in a healthy state, taking into account the prevalence of different health conditions and mortality, etc. (7). Healthy expectancy is a composite indicator that shifts the focus from the quantity of life to the quality of life (8). In the context of the aging global population and the high incidence of chronic diseases, mortality indicators alone are no longer sufficient to reflect the health of the population, and the application of HLE is becoming increasingly widespread.

Since the 1980's, three broad hypotheses have been proposed for the future course of mortality and morbidity. A theory of “expansion of morbidity” proposed by Gruenberg (9) assumes that the increase in LE is due to a reduction in the mortality of chronic diseases rather than a reduction in the incidence of these diseases. Therefore, the prolongation of life span should coexist with a growing number of years in poor health. In contrast, Fries (10) proposed the “compression of morbidity” hypothesis, which asserts that prevention, improvement in living conditions, and healthier lifestyles will reduce the time spent with chronic diseases and disabilities before death. Manton (11) proposed a third theory, “dynamic equilibrium,” which combined elements of both the compression and expansion hypotheses. He viewed the reduction in mortality as at least partly the result of lower rates of chronic disease progression. Since the decline in disease progression delayed the emergence of more severe disease states, the dynamic balance scenario meant that the reduction in mortality would be associated with the redistribution of disease and disability from more severe states to less severe states. In this case, the proportion of LE with severe illness or disability stabilizes or decreases, while the proportion of LE with moderate disability or less severe illness increases. These three situations put different pressures on health services and systems, and incorrect judgment can lead to inappropriate directions for health policy and waste of health resources.

Due to the diversity of health concepts, different health meanings correspond to different healthy expectancies. This gave rise to more specific terms for HLE, such as disability-free LE (6) and dementia-free LE (12). In this paper, we used self-rated health (SRH) to calculate HLE. The use of SRH has both strengths and weaknesses. In terms of strengths, SRH is a simple and important evaluation indicator that can not only reflect personal health status but also integrate the subjective and objective aspects of health status (13). SRH can not only measure current level of health but also measure changes in health and health cognition, and it is an independent predictor of mortality (14, 15). The comprehensiveness of SRH indicators makes people more likely to assess their health holistically, considering various social, physical, and emotional factors that affect their health. In terms of weakness, the main defect of SRH lies in its subjective nature. SRH is conducted by the respondents themselves in an extensive and subjective assessment, and it is different from health as evaluated by a doctor. Therefore, SRH may be more susceptible to external factors such as gender, race, and income level, as well as changes in attitudes and expectations about health over time (8).

To date, there are few studies on HLE in China, especially regarding SRH as the evaluation standard. Among them, the only study with residents of Jiangxi as the research subjects is our recent study on the LE of elderly people aged 60 and older in Jiangxi without dementia (12). Two other studies on LE without disabilities in various provinces in China mentioned Jiangxi but did not discuss Jiangxi alone (16, 17). The above three studies of HLE are not based on SRH. There are also studies on other individual provinces, such as Shanghai (18). Because the “rates” used in various studies (such as SRH rates and disability rates) or target age groups are different, there are problems with the comparability of the results of the studies. Despite the comparability problem, we have found some common themes in these studies. Women have longer LE than men, but their HLE/LE ratio is lower than that of men, which indicates that their quality of life is lower than that of men. This is consistent with some studies in other countries, such as Japan and South Korea (19), Thailand (20), the United States (21), and Finland (22). This common phenomenon is called the female–male health–survival paradox (23). It has been widely recognized by the academic community (24–28). Many explanations of this paradox are rooted in biological, sociological, and psychological explanations. There are likely a variety of reasons, including basic biological differences between genders, such as genetic factors, immune system responses, hormones, and disease patterns. Differences in behavior, such as risk taking and unwillingness to seek and follow treatment, may also play a role (23).

In the study of Zhou et al. (17), both LE and HLE (disability-free LE) increased substantially in China from 1990 to 2015. In 2015, LE and HLE at birth were 76.2 and 68.0 years old, respectively, 9.5 years and 8.4 years older than 1990. However, the increase in LE was greater than that of HLE at both the national level and provincial level, indicating an expansion of morbidity from 1990 to 2015. A Japanese study using SRH to compute health expectancy showed that Japanese individuals first experienced a process of morbidity compression from 1986 to 1995 and then experienced morbidity expansion (8). The emergence of this trend seemed to be related to the implementation of Japanese policies and economic conditions in the same period. A study in Thailand on self-rated healthy life expectancy (SRHLE) showed a compression of morbidity from 1986 to 1995 (29). Another study in Thailand on SRHLE and disability-free LE showed that it experienced a period of expansion of incidence from 2002 to 2007 (20). Some other countries and regions have experienced an expansion of morbidity (30–32), while others indicate a compression of morbidity (33–36).

Our study was the first to calculate the HLE of Jiangxi based on SRH. It will fill the gap of SRHLE in Jiangxi. The objective of this paper was to calculate the LE and SRHLE of urban–rural male–female residents in Jiangxi in 2013 and 2018 and to estimate their trends. At the same time, we study the difference in LE and SRHLE between genders and between urban–rural areas and explore the trends in these differences. Our study presents a useful way to comprehensively assess the health level of residents in Jiangxi and provide a reference for the formulation of relevant health policies. At the same time, it can also serve as a reference for other countries and regions.

## Materials and Methods

### SRH Data

The SRH data were derived from the 2013 and 2018 National Health Services Survey (NHSS), which has been organized by the National Health Commission of the People's Republic of China every 5th year since 1993. Multistage stratified cluster random sampling was used in NHSS (37, 38). The first stage sampled counties/districts; the second sampled townships/streets; the third stage randomly sampled two villages (rural areas) or residents' committees (urban areas) in each township or street; the last stage randomly sampled 60 households in selected villages/residents' committees, where respondents were actual members of the households selected. All households in the sample village are numbered. The sampling interval is equal to the number of households divided by 60 (rounded to the whole). The first household is determined randomly, the second household is the number of the first household plus the sampling interval, and so on. The surveys encompassed all 31 provinces in mainland China. In Jiangxi (China), Donghu District, Zhanggong District, Yuanzhou District, Shanggao County, Gao'an County, and Poyang County were selected as the sample counties (cities, districts) representing the overall situation of urban and rural areas in Jiangxi (12). The sample counties (districts) selected in the two surveys were the same. The 2013 survey included 3,600 households and 11,252 people; the 2018 survey included 3,600 households and 10,123 people. Face-to-face interviews were conducted by uniformly trained investigators.

The EuroQol-visual analog scale (EQ-VAS) was used for SRH. Those who participated in the self-rated health survey in 2013 were at age 15 and older, while those in 2018 were 10 and older. For comparison, we only counted the SRH status of those 15 and older. The numbers of residents aged 15 and older in the surveys in 2013 and 2018 were 8,797 and 7,916, respectively. In these two surveys, all residents aged 15 and older underwent self-rated health surveys. The reason why the response rate is so high is that the survey adopts face-to-face inquiry at home and is based on a simple SRH question, not based on physical health examination. The VAS consisted of a horizontal 11-cm line where every centimeter was marked and labeled 0, 10, 20, 100, with anchor points 0 (worst health state) and 100 (best health state). The question was “Please state the score that best represents your health condition today.” A study by Perneger et al. (39) suggested that the EQ-VAS scale and SRH (with answer categories, e.g., excellent/very good/good/fair/poor) are practically equivalent in terms of the measured health construct. The EQ-VAS scale may prove more consistent across socioeconomic groups, education levels, and possibly languages since it only needs health descriptors at the extremities of the scale. In this study, people with SRH scores of 60 and above were classified as healthy, and the overall SRH proportion of people aged 15 and above was calculated by age.

### Mortality Data and Calculation of LE

The infant mortality rate (IMR) and under 5 mortality rate (U5MR) were gathered from the Summary of Health Statistics in Jiangxi (2013 and 2018) (40, 41), which were compiled annually by the Health Commission of Jiangxi and used internally. The IMR and U5MR data come from the national maternal and child health monitoring system. The China model life table (42) was used to compile the life table of Jiangxi in 2013 and 2018 by gender and urban–rural area. The principle of the China model life table is similar to that of the Murray model life table (43). However, the parameters of the China model life table are generated based on China's original data, including data from China's three censuses (1990, 2000, and 2010) and two populations from 1% sample surveys (1995 and 2005). The China model life table is the same as the Murray model life table, and the whole life table can be estimated by using one parameter (such as U5MR) or two parameters (such as U5MR and adult mortality) (43).

### Sullivan Method

Sullivan method was used to compute SRHLE (44). The method was based on the current life table and the prevalence of SRH of the population obtained from the cross-sectional survey data, subdivides the number of surviving person-years in the life table, and finally decomposes the LE into self-rated healthy (or unhealthy) LE. The European Health Expectancy Monitoring Unit (EHEMU) published the latest practice guide (fourth edition) for the computation of HLE based on Sullivan's method (44). This study uses the method recommended by the guide to measure HLE; the formula is as follows:

(1) $$\begin{array}{l} HLE_{x}=\frac{1}{l_{x}}\overset{x_{\max}}{\underset{x}{\sum }}\left(\;L_{x}\times \;\pi_{x}\right) \end{array}$$

In the above formula, *l*_*X*_ and *L*_*X*_ are the number of survivors and the survival person-years in the corresponding age group in the life table, respectively. π_*X*_ is a newly added indicator item in the life table, which represents the prevalence of SRH in the population of different age groups. The standard error in the above formula can be approximated by the following formula:

(2) $$\begin{array}{l} S\;\left(HLE_{x}\right)\;\approx \;\sqrt{\frac{1}{l_{x}^{2}}\overset{x_{\max}}{\underset{x}{\sum }}L_{x}^{2}\frac{\pi_{x}\;\left(\;1\;-\;\pi_{x}\;\right)}{N_{x}}} \end{array}$$

The 95% confidence interval of *HLE*_*x*_ is equal to *HLE*_*x*_ ± *S*(*HLE*_*x*_) × 1.96. There is a detailed calculation process in the Sullivan method guide (44) for calculating the standard error and confidence interval of HLE. The changes and differences in residents' health levels were evaluated by comparing the absolute differences in LE and SRHLE in different years, urban–rural areas, and genders. The proportion of SRHLE on LE was used to evaluate the quality of LE. In addition, we applied the general algorithm proposed by Andreev., et al. (45) to decompose changes in SRHLE into mortality and health effects.

The significance level was set at α = 0.05. Statistical analyses were performed using SPSS 25.0. Continuous variables are presented as the mean ± SD.

## Results

### SRH Rates

**Figure 1:**
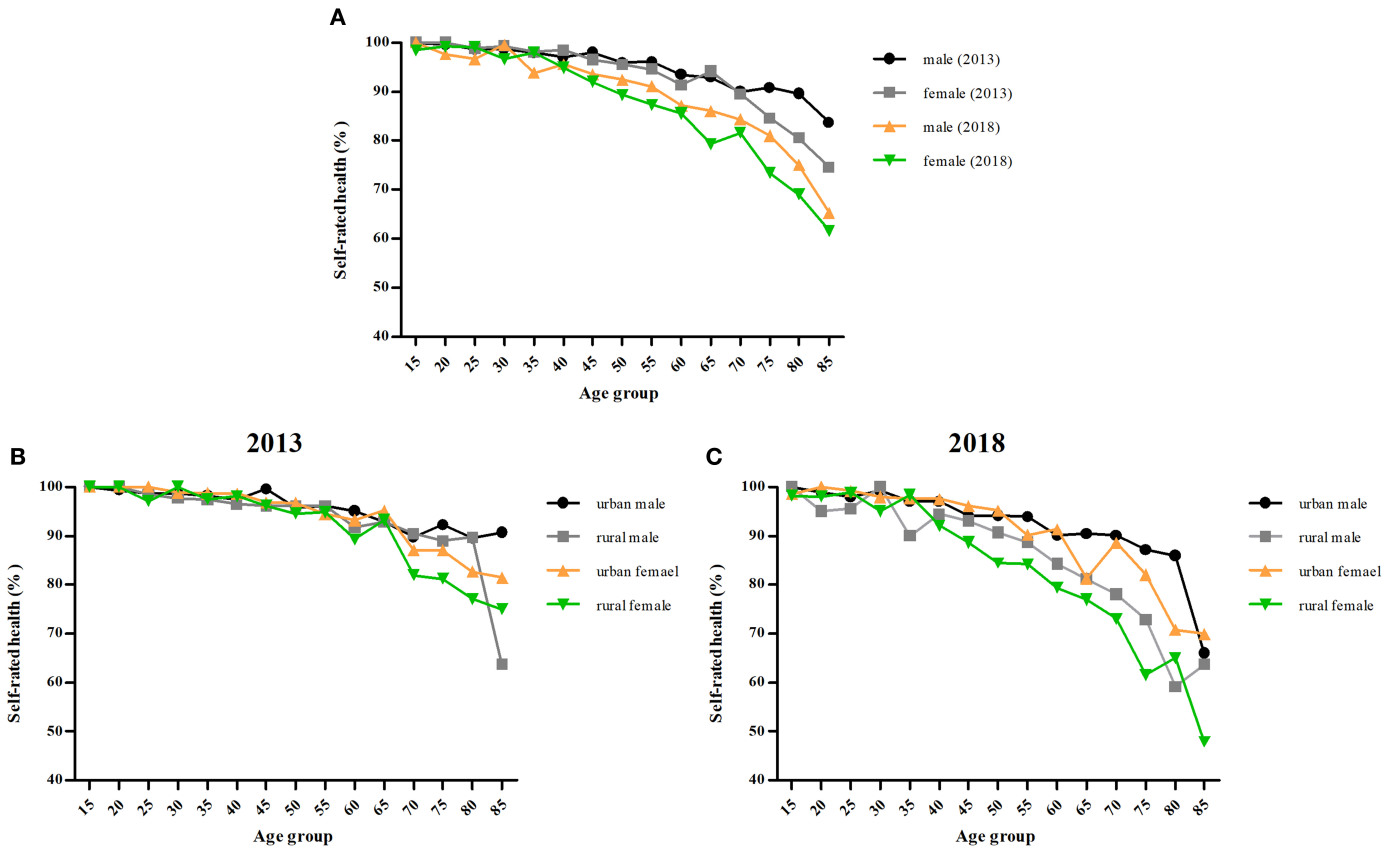
Trends in SRH rates of males–females. **(A)** Trends in SRH rates in 2013 and 2018. **(B)** Trends in SRH rates of urban–rural residents in 2013. **(C)** Trends in SRH rates of urban–rural residents in 2018.

The SRH scores of residents aged 15 and above were 80.70 ± 12.81 and 76.23 ± 15.98 in 2013 and 2018, respectively. The trends in SRH rates (%) are presented in Figure 1. The surveys of residents in Jiangxi (China) in 2013 and 2018 show that with increasing age, SRH rates decrease gradually for both men and women, from nearly 100% for persons aged 15 to <80% at age 80 in 2013 and at age 60 in 2018. Comparing 2013 and 2018, the total SRH rates decreased from 96.01% and 95.33% in 2013 for men and women, respectively, to 91.36% and 89.09% in 2018. Men tend to rate their health better than women overall. The total SRH rates of men were 0.68% (χ^2^= 2.868, *P* > 0.05) and 2.27% (χ^2^ = 12.484, *P* < 0.01) higher than those of women in 2013 and 2018, respectively. Urban residents' SRH rates were higher than those of rural residents in both 2013 and 2018. The total SRH rates for urban residents of men were 1.12% (χ^2^ = 3.720, *P* > 0.05) and 4.44% (χ^2^ = 32.265, *P* < 0.01) higher than those for rural residents in 2013 and 2018, respectively, and those for urban women were 1.53% (χ^2^ = 6.818, *P* < 0.01) and 5.51% (χ^2^ = 14.438, *P* < 0.01) higher than those for rural residents.

### Trend in LE and SRHLE by Age

For all age groups, LE increased between 2013 and 2018, but the SRH rates decreased. Were the residents of Jiangxi living longer but in a state of poor health?

**Figure 2:**
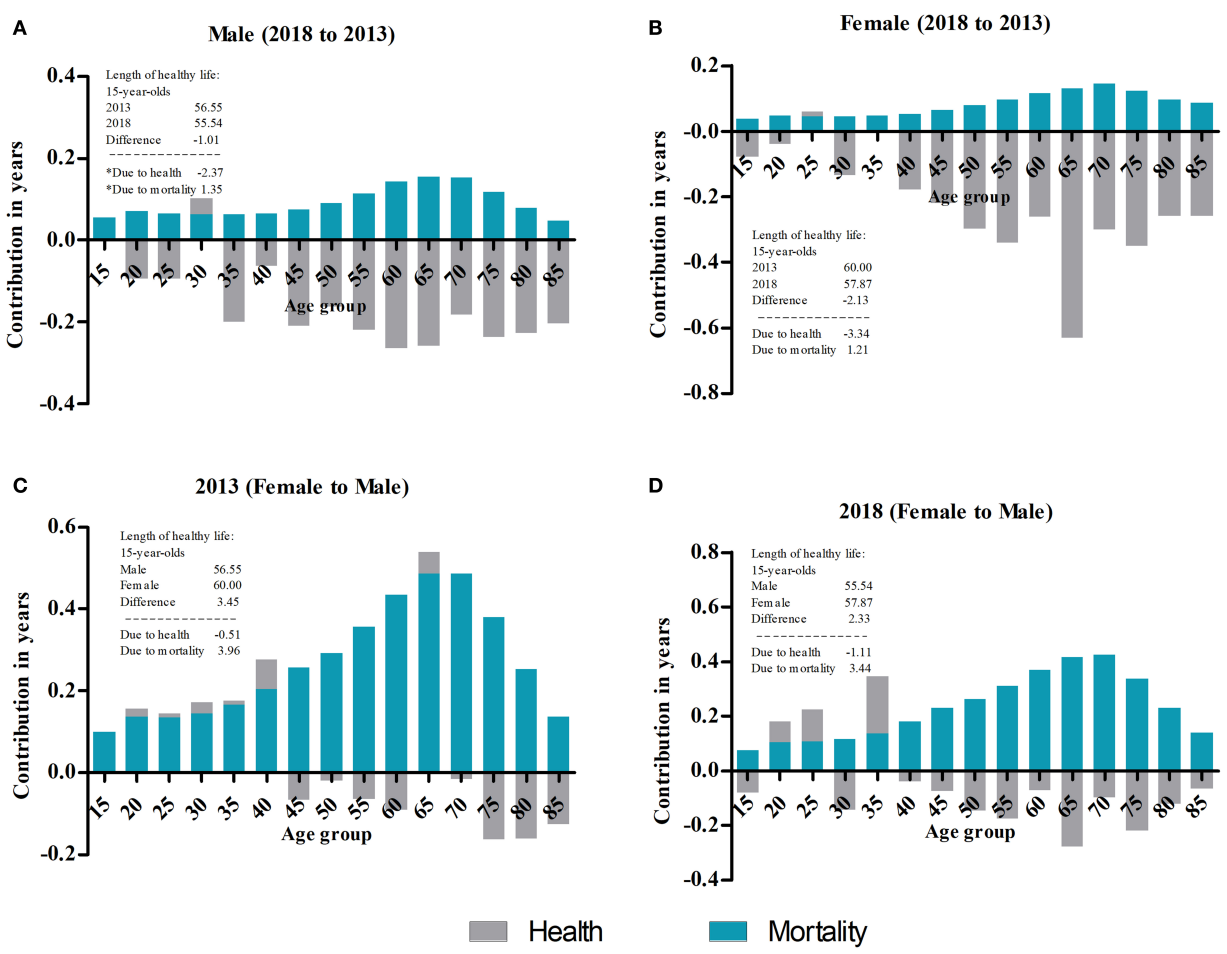
Decomposition of the difference in SRHLE. **(A)** Decomposition of the difference between male SRHLE at age 15 between 2013 and 2018. *Due to rounding, the addition does not equal the exact SRHLE difference. **(B)** Decomposition of the difference between female SRHLE at age 15 between 2013 and 2018. **(C)** Decomposition of the difference between 2013 SRHLE at age 15 between males and females. **(D)** Decomposition of the difference between 2018 SRHLE at age 15 between males and females.

Tables 1, 2 show LE and SRHLE for people aged 15 and older based on data from the 2013 and 2018 surveys. LE increased for every age group, from 58.78 to 60.35 years at age 15 and from 4.81 to 5.13 years at age 85 for men and from 63.26 to 64.78 years at age 15 and from 5.49 to 5.85 years at age 85 for women. However, SRHLE decreased for every age group, from 56.55 to 55.54 years at age 15 and from 4.06 to 3.35 years at age 85 for men; from 60.00 to 57.87 years at age 15 and from 4.09 to 3.60 years at age 85 for women. All age groups had statistically significant differences in HLE between 2013 and 2018 except for males aged 65, 70, 80, and 85 and females aged 70 and above. The statistical significance of the difference can be judged by comparing whether the confidence interval of SRHLE has an intersection (Tables 1, 2) or whether the confidence interval of the absolute SRHLE difference between the groups contains zero (Figure A1). As expected, the proportion of SRHLE also decreased for every age group, from 96.21 to 92.31% at age 15 and from 83.73 to 65.28% at age 85 for men; from 94.85 to 89.34% at age 15 and from 74.52 to 61.56% at age 85 for women. The proportion of SRHLE on LE decreased with increased age. Figures 2A,B show the decomposition of the difference between men's and women's SRHLE at age 15 between 2013 and 2018. This suggests that −2.37 years of the overall difference of −1.01 (2018 to 2013) years is attributable to differences in health, and 1.35 years is attributable to differences in mortality for men. The contribution of health is greater than that of mortality, and the maximum age-specific contributions are produced for men aged 60 and 65. In the overall difference of −2.13 years among women, −3.34 years are attributed to health and 1.21 years are attributed to mortality. The maximum age-specific contributions are produced for women aged 65.

**Table 1:** LE and SRHLE of Jiangxi Province of China in 2013.

**Age**	**Male**				**Female**			
	**LE**	**SRHLE and 95% CI**	**SE**	**% of LE**	**LE**	**SRHLE and 95% CI**	**${\text{SE}}_{\text{SRHLE}}^{\text{a}}$**	**% of LE**
**Total**								
15–19	58.78	56.55 (56.22–56.88)	0.170	96.21	63.26	60.00 (59.56–60.44)	0.225	94.85
20–24	54.01	51.77 (51.44–52.10)	0.171	95.86	58.39	55.13 (54.69–55.57)	0.226	94.41
25–29	49.29	47.06 (46.73–47.39)	0.170	95.48	53.55	50.28 (49.84–50.72)	0.226	93.89
30–34	44.58	42.41 (42.08–42.74)	0.168	95.13	48.72	45.49 (45.05–45.93)	0.225	93.38
35–39	39.89	37.77 (37.45–38.09)	0.165	94.67	43.90	40.70 (40.26–41.14)	0.225	92.71
40–44	35.27	33.23 (32.91–33.55)	0.162	94.22	39.12	36.00 (35.56–36.44)	0.223	92.02
45–49	30.74	28.83 (28.52–29.14)	0.159	93.78	34.41	31.34 (30.90–31.78)	0.223	91.08
50–54	26.36	24.51 (24.2–24.820)	0.159	92.98	29.80	26.87 (26.43–27.31)	0.222	90.17
55–59	22.15	20.45 (20.14–20.76)	0.156	92.31	25.33	22.57 (22.14–23.00)	0.221	89.11
60–64	18.16	16.58 (16.27–16.89)	0.157	91.27	21.03	18.46 (18.03–18.89)	0.222	87.78
65–69	14.53	13.15 (12.84–13.46)	0.159	90.51	17.01	14.75 (14.32–15.18)	0.222	86.72
70–74	11.32	10.12 (9.8–10.44)	0.163	89.37	13.34	11.18 (10.73–11.63)	0.230	83.78
75–79	8.66	7.71 (7.38–8.04)	0.169	88.97	10.19	8.23 (7.75–8.71)	0.242	80.72
80–84	6.45	5.63 (5.24–6.02)	0.199	87.23	7.53	5.85 (5.32–6.38)	0.273	77.76
85+	4.85	4.06 (3.53–4.59)	0.273	83.73	5.49	4.09 (3.43–4.75)	0.335	74.52
**Urban**								
15–19	60.93	58.79 (58.32–59.26)	0.239	96.49	65.47	62.35 (61.71–62.99)	0.327	95.23
20–24	56.08	53.94 (53.47–54.41)	0.239	96.18	60.55	57.42 (56.78–58.06)	0.327	94.84
25–29	51.27	49.16 (48.69–49.63)	0.237	95.87	55.64	52.51 (51.87–53.15)	0.328	94.37
30–34	46.48	44.42 (43.96–44.88)	0.234	95.57	50.74	47.61 (46.97–48.25)	0.328	93.82
35–39	41.71	39.71 (39.26–40.16)	0.230	95.20	45.86	42.78 (42.14–43.42)	0.326	93.27
40–44	37.01	35.08 (34.63–35.53)	0.227	94.80	41.02	37.99 (37.35–38.63)	0.325	92.61
45–49	32.40	30.59 (30.15–31.03)	0.223	94.41	36.23	33.26 (32.62–33.90)	0.325	91.78
50–54	27.93	26.11 (25.67–26.55)	0.226	93.49	31.53	28.69 (28.06–29.32)	0.323	90.98
55–59	23.61	21.96 (21.53–22.39)	0.220	92.99	26.95	24.23 (23.60–24.86)	0.322	89.92
60–64	19.49	17.97 (17.54–18.40)	0.218	92.20	22.51	20.01 (19.38–20.64)	0.320	88.91
65–69	15.69	14.32 (13.89–14.75)	0.220	91.25	18.32	16.07 (15.44–16.70)	0.321	87.71
70–74	12.29	11.13 (10.70–11.56)	0.218	90.54	14.48	12.31 (11.66–12.96)	0.330	85.03
75–79	9.43	8.58 (8.17–8.99)	0.210	91.03	11.11	9.34 (8.68–10.00)	0.336	84.09
80–84	7.03	6.33 (5.87–6.79)	0.236	90.04	8.25	6.77 (6.04–7.50)	0.375	82.10
85+	5.25	4.76 (4.23–5.29)	0.271	90.62	6.04	4.92 (4.04–5.80)	0.451	81.48
**Rural**								
15–19	58.56	55.89 (55.33–56.45)	0.285	95.44	63.02	59.07 (58.36–59.78)	0.360	93.74
20–24	53.79	51.11 (50.55–51.67)	0.287	95.02	58.16	54.2 (53.49–54.91)	0.361	93.20
25–29	49.09	46.39 (45.83–46.95)	0.288	94.51	53.32	49.35 (48.64–50.06)	0.362	92.56
30–34	44.38	41.75 (41.19–42.31)	0.285	94.07	48.50	44.66 (43.96–45.36)	0.356	92.09
35–39	39.71	37.17 (36.63–37.71)	0.275	93.61	43.69	39.83 (39.13–40.53)	0.358	91.18
40–44	35.09	32.66 (32.13–33.19)	0.270	93.08	38.92	35.18 (34.48–35.88)	0.355	90.38
45–49	30.57	28.29 (27.77–28.81)	0.266	92.53	34.22	30.54 (29.84–31.24)	0.355	89.24
50–54	26.20	24.06 (23.54–24.58)	0.264	91.83	29.62	26.08 (25.39–26.77)	0.354	88.06
55–59	22.00	19.99 (19.47–20.51)	0.263	90.86	25.16	21.83 (21.14–22.52)	0.354	86.77
60–64	18.03	16.11 (15.58–16.64)	0.270	89.40	20.87	17.70 (17.00–18.40)	0.358	84.82
65–69	14.41	12.77 (12.23–13.31)	0.277	88.57	16.87	14.08 (13.37–14.79)	0.360	83.45
70–74	11.23	9.72 (9.13–10.31)	0.299	86.54	13.23	10.53 (9.79–11.27)	0.377	79.60
75–79	8.59	7.21 (6.55–7.87)	0.338	83.99	10.10	7.91 (7.17–8.65)	0.379	78.36
80–84	6.40	5.07 (4.23–5.91)	0.430	79.26	7.46	5.68 (4.86–6.50)	0.416	76.15
85^b^+	4.81	3.06 (1.69–4.43)	0.698	63.65	5.44	4.08 (3.14–5.02)	0.481	75.02

^a^*SE, Standard error*.

b *The 85+ group is the open interval*.

**Table 2:** LE and SRHLE of Jiangxi Province of China in 2018.

**Age**	**Male**				**Female**			
	**LE**	**SRHLE and 95% CI**	**SE_**SRHLE**_**	**% of LE**	**LE**	**SRHLE and 95% CI**	**SE_**SRHLE**_**	**% of LE**
**Total**								
15–19	60.35	55.54 (55.05–56.03)	0.249	92.03	64.78	57.87 (57.28–58.46)	0.300	89.34
20–24	55.52	50.70 (50.21–51.19)	0.250	91.31	59.87	53.04 (52.46–53.62)	0.295	88.59
25–29	50.74	46.01 (45.54–46.48)	0.242	90.67	54.98	48.18 (47.61–48.75)	0.293	87.62
30–34	45.96	41.37 (40.91–41.83)	0.234	90.02	50.10	43.33 (42.76–43.9)	0.292	86.47
35–39	41.21	36.63 (36.17–37.09)	0.234	88.87	45.24	38.61 (38.05–39.17)	0.288	85.35
40–44	36.53	32.22 (31.79–32.65)	0.219	88.19	40.42	33.86 (33.3–34.42)	0.285	83.79
45–49	31.95	27.80 (27.38–28.22)	0.215	87.02	35.65	29.32 (28.77–29.87)	0.282	82.24
50–54	27.50	23.60 (23.19–24.01)	0.210	85.83	30.98	24.98 (24.44–25.52)	0.277	80.65
55–59	23.21	19.59 (19.19–19.99)	0.206	84.40	26.43	20.88 (20.35–21.41)	0.273	79.00
60–64	19.13	15.80 (15.40–16.20)	0.203	82.60	22.03	16.98 (16.46–17.50)	0.267	77.09
65–69	15.37	12.46 (12.07–12.85)	0.199	81.09	17.89	13.36 (12.84–13.88)	0.265	74.69
70–74	12.02	9.48 (9.09–9.87)	0.201	78.87	14.10	10.29 (9.77–10.81)	0.265	73.00
75–79	9.21	6.96 (6.55–7.37)	0.209	75.58	10.80	7.42 (6.89–7.95)	0.270	68.72
80–84	6.86	4.86 (4.42–5.30)	0.226	70.84	8.01	5.24 (4.68–5.80)	0.288	65.42
85+	5.13	3.35 (2.79–3.91)	0.288	65.28	5.85	3.60 (2.91–4.29)	0.353	61.56
**Urban**								
15–19	61.80	58.17 (57.58–58.76)	0.302	94.12	66.30	61.06 (60.32–61.80)	0.376	92.10
20–24	56.93	53.29 (52.70–53.88)	0.303	93.60	61.37	56.20 (55.48–56.92)	0.370	91.58
25–29	52.10	48.5 (47.91–49.09)	0.299	93.09	56.44	51.27 (50.54–52.00)	0.370	90.83
30–34	47.27	43.77 (43.2–44.34)	0.290	92.58	51.53	46.38 (45.66–47.10)	0.368	90.02
35–39	42.48	38.99 (38.42–39.56)	0.289	91.80	46.62	41.58 (40.87–42.29)	0.364	89.17
40–44	37.74	34.38 (33.83–34.93)	0.279	91.10	41.76	36.81 (36.11–37.51)	0.360	88.15
45–49	33.10	29.87 (29.33–30.41)	0.273	90.22	36.95	32.10 (31.40–32.80)	0.357	86.88
50–54	28.60	25.6 (25.08–26.12)	0.265	89.54	32.21	27.53 (26.84–28.22)	0.353	85.46
55–59	24.24	21.47 (20.96–21.98)	0.259	88.56	27.59	23.09 (22.40–23.78)	0.350	83.68
60–64	20.07	17.50 (17.00–18.00)	0.253	87.21	23.10	19.00 (18.33–19.67)	0.343	82.25
65–69	16.20	13.98 (13.50–14.46)	0.245	86.29	18.86	15.05 (14.38–15.72)	0.342	79.82
70–74	12.73	10.76 (10.28–11.24)	0.246	84.58	14.95	11.85 (11.19–12.51)	0.338	79.30
75–79	9.78	7.96 (7.46–8.46)	0.255	81.47	11.50	8.63 (7.95–9.31)	0.348	75.01
80–84	7.31	5.62 (5.08–6.16)	0.276	76.94	8.57	6.03 (5.30–6.76)	0.375	70.39
85+	5.45	3.60 (2.88–4.32)	0.365	66.04	6.28	4.40 (3.51–5.29)	0.455	69.99
**Rural**								
15–19	60.08	53.71 (52.87–54.55)	0.429	89.40	64.50	55.36 (54.38–56.34)	0.500	85.83
20–24	55.26	48.87 (48.03–49.71)	0.430	88.44	59.60	50.54 (49.57–51.51)	0.493	84.79
25–29	50.49	44.32 (43.54–45.10)	0.396	87.79	54.72	45.74 (44.79–46.69)	0.484	83.59
30–34	45.72	39.75 (39.00–40.50)	0.382	86.94	49.85	40.90 (39.96–41.84)	0.482	82.06
35–39	40.98	34.98 (34.23–35.73)	0.385	85.34	44.99	36.27 (35.34–37.20)	0.473	80.62
40–44	36.31	30.75 (30.06–31.44)	0.354	84.70	40.18	31.50 (30.58–32.42)	0.471	78.41
45–49	31.74	26.39 (25.71–27.07)	0.347	83.16	35.42	27.09 (26.18–28.00)	0.464	76.49
50–54	27.30	22.21 (21.54–22.88)	0.343	81.34	30.75	22.91 (22.01–23.81)	0.457	74.49
55–59	23.03	18.25 (17.58–18.92)	0.339	79.27	26.22	19.03 (18.14–19.92)	0.452	72.59
60–64	18.96	14.55 (13.89–15.21)	0.337	76.73	21.84	15.26 (14.39–16.13)	0.445	69.89
65–69	15.22	11.29 (10.63–11.95)	0.337	74.15	17.72	11.91 (11.04–12.78)	0.442	67.18
70–74	11.90	8.45 (7.78–9.12)	0.344	71.00	13.96	8.87 (7.99–9.75)	0.448	63.56
75–79	9.11	6.07 (5.35–6.79)	0.366	66.64	10.68	6.28 (5.38–7.18)	0.457	58.80
80–84	6.79	4.15 (3.36–4.94)	0.403	61.09	7.91	4.50 (3.54–5.46)	0.490	56.86
85+	5.08	3.23 (2.21–4.25)	0.521	63.66	5.78	2.77 (1.64–3.90)	0.578	47.96

### Gender Differences in LE and SRHLE

Both the LE and SRHLE for women were higher than those for men in all age groups, but the proportion of SRHLE on LE was lower than that of men. This trend was found in both 2013 and 2018 (Tables 1, 2). The LE and SRHLE of women at age 15 were 4.48 and 3.45 years higher than those of men in 2013, respectively, and those of women at age 85 were 0.64 and 0.04 years higher than men; these differences were 4.43 and 2.34 years, and 0.72 and 0.25 years in 2018, respectively. The absolute differences in SRHLE between men and women were smaller than the absolute difference in LE for all age groups, and both decreased with increasing age. There were statistically significant differences in SRHLE between men and women for all age groups in 2013 and 2018, except for age groups 75 and above in 2013 and 65 and above in 2018. The proportions of SRHLE in 2013 for men were 96.21 and 83.73% at ages 15 and 85, respectively, and were 94.85 and 74.52% for women; for men in 2018, the proportions were 92.03 and 65.28% at ages 15 and 85, respectively, and 89.34 and 61.56% for women. Figures 2C,D show the decomposition of the difference between 2013 and 2018 SRHLE at age 15 between men and women. This suggests that −0.51 years of the overall difference of 3.45 (female to male) years is attributable to differences in health, and 3.96 years are attributable to differences in mortality in 2013. The contribution of mortality is greater than that of health, and the maximum age-specific contributions are produced for persons aged 65 and 70. In the overall difference of 2.33 years in 2018, −1.11 years are attributed to health and 3.44 years are attributed to mortality. The maximum age-specific contributions are produced for people aged 65 and 70.

### Urban–Rural Differences in LE and SRHLE

**Figure 3:**
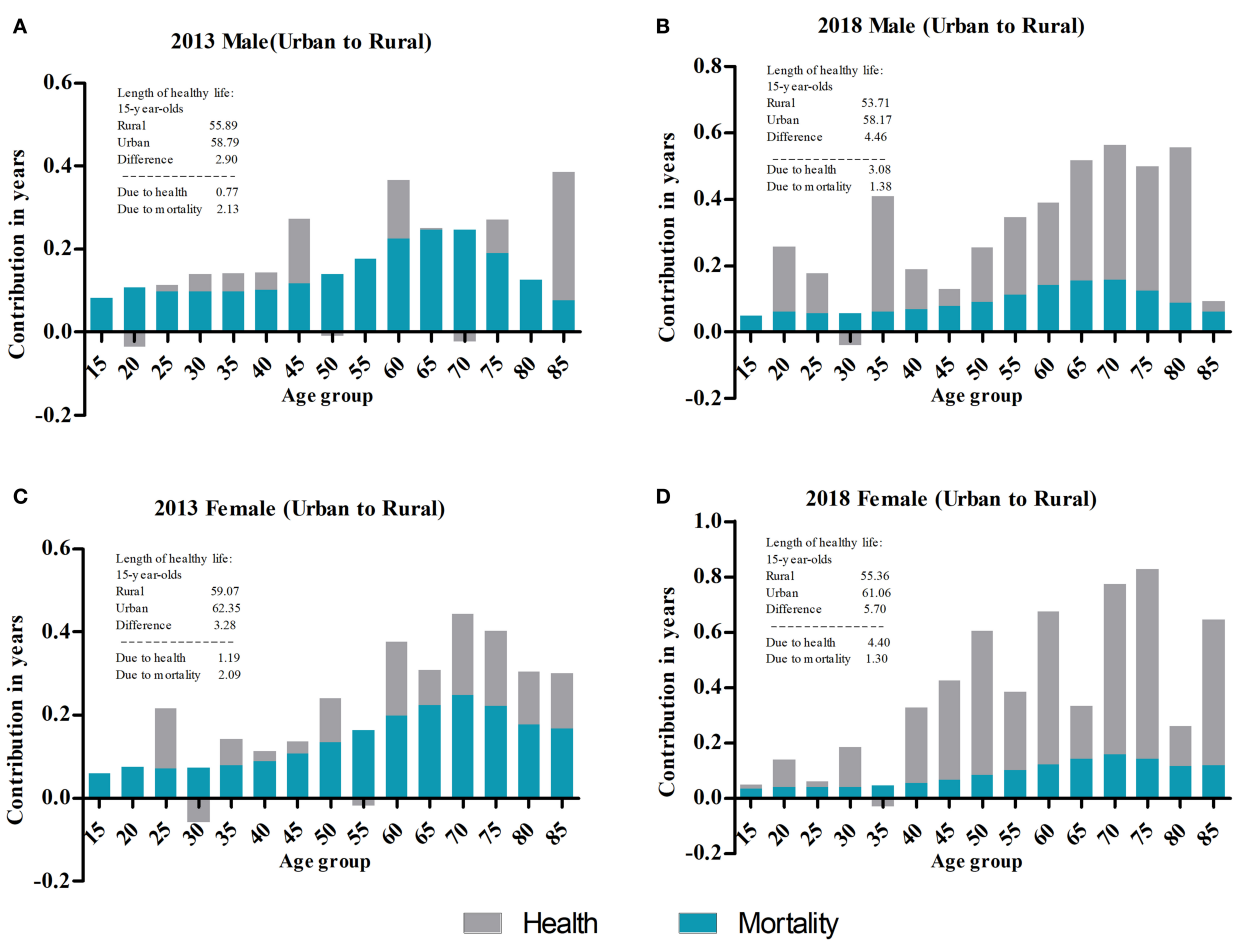
Decomposition of the difference in SRHLE. **(A)** Decomposition of the difference between male SRHLE at age 15 between urban and rural areas in 2013. **(B)** Decomposition of the difference between male SRHLE at age 15 between urban and rural areas in 2018. **(C)** Decomposition of the difference between female SRHLE at age 15 between urban and rural areas in 2013. **(D)** Decomposition of the difference between female SRHLE at age 15 between urban and rural areas in 2018.

Both the LE and SRHLE of urban residents for all age groups were higher than those of rural residents, and the proportion of SRHLE was higher than that of rural residents in 2013 and 2018 (Tables 1, 2). The LE and SRHLE of male urban residents at age 15 in 2013 were 2.37 and 2.90 years higher than rural residents, respectively, and were 0.44 and 1.70 years at age 85; female urban residents at age 15 were 2.45 and 3.28 years higher than rural residents, respectively, and were 0.60 and 0.48 years at age 85. The LE and SRHLE of male urban residents at age 15 in 2018 were 1.72 and 4.46 years higher than rural residents, respectively, and were 0.37 and 0.36 years at age 85; female urban residents at age 15 were 1.80 and 5.57 years higher than rural residents, respectively, and were 0.50 and 1.63 years at age 85. There were statistically significant differences in HLE between urban and rural areas in all age groups for both genders in 2013 and 2018 except for both genders for the age groups 80 and above in 2013, women 80 and above and men 85 and above in 2018. The absolute differences between urban and rural SRHLE for almost all age groups were higher than the absolute differences in LE, and both decreased with increasing age. Figure 3 shows the decomposition of the difference in urban–rural SRHLE by gender in 2013 and 2018. The contribution of health in 2013 was less than the contribution of mortality, while in 2018, it was the opposite.

### SRHLE Under Different SRH Standard: Considering the Subjectivity of SRH

The SRHLE of residents in Jiangxi (China) under different SRH standards (≥70, ≥80, and ≥90) was calculated to verify whether the differences in SRHLE between years, gender, and urban–rural areas would change due to changes in SRH standards. For year differences, the absolute differences in SRHLE (2018 to 2013) for people aged 15 were all less than zero under different SRH standards (≥70, ≥80, and ≥90), which was the same as when the SRH standard was ≥60. The SRHLE of residents in Jiangxi decreased in 2018 compared with 2013. For urban–rural differences, the absolute differences in SRHLE (urban to rural) for persons aged 15 were all greater than zero under different SRH standards (≥70, ≥80, and ≥90) in both 2013 and 2018, which was also the same as when the SRH standard was ≥60. The SRHLE of urban residents is higher than that of rural residents. For gender differences, the SRHLE of women is gradually lower than that of men for persons aged 15 as the SRH standard increases, which is contrary to the results under the SRH standard of ≥60. The absolute gender difference of SRHLE (female to male) is greater than zero under the SRH standard of 60, while as the SRH standard increases, the difference is gradually less than zero. However, although women have higher SRHLE than men under the SRH standard of ≥60, their proportion of SRHLE is lower than that of men, which indicates that the quality of LE in women is lower than that in men. With the improvement of SRH standards, women's disadvantages in the proportion of SRHLE on LE are directly shown as disadvantages in SRHLE. Detailed results are shown in the Appendix of this article.

## Discussion

This paper has examined the LE and SRHLE of urban–rural men and women in Jiangxi (China) from 2013 to 2018. Our results show that both genders enjoyed an increase in LE but a decrease in SRHLE from 2013 to 2018. The analysis of the SRH rate indicated that the SRH status of both genders in 2018 was worse than that in 2013. The relevant studies considered that changes in LE accompanied by a decreased proportion of healthy years can be evidence of an expansion of morbidity (46). Our study supports the hypothesis of an expansion of morbidity (9), which is consistent with the 1995–2004 situation in Japan (8) and the 2002–2007 situation in Thailand (20) and other countries and regions (30–32). This shows that the change in health status is a more complex and comprehensive manifestation. It cannot be assumed that the health status of residents is improving year by year because of the year-on-year increase in LE.

The increased LE was the result of decreased mortality, and the primary reason for the decreased SRHLE was the decreased rate of SRH. Therefore, it was important to determine the reasons for the decreased SRH rates. In the Thai study, the compression of morbidity in the period 1986–1995 and expansion in 2002–2007 were thought to be caused by economic reasons. Thailand experienced a “soap bubble” economic period in 1986–1995, and the economy developed rapidly, but it slowed down in 2002–2007 (20). In the Japanese study, the compression of morbidity in the period 1986–1995 was explained in relation to the background factors of policy implementation and Japan's economic conditions during the same period. In 1989, the Japanese government implemented the Golden Plan to promote health and welfare services for the elderly. From 1986 to 1990, Japan also experienced a bubble economy period. The expansion of morbidity was explained in relation to the 1995–2004 Asian financial crisis (8). Studies have shown that a decline in self-rated health is causally related to concerns about financial difficulties and work insecurity (47, 48). Jiangxi also experienced a slowdown in economic development from 2013 to 2018 (49). This may be one of the reasons for the decreased SRHLE of residents in Jiangxi.

SRH can not only reflect personal health status but also integrate the subjective and objective aspects of health status (13). Poor SRH may be caused by an actual health condition and may also be caused by a common feature of negative psychosocial conditions (such as outliers, negative life events, depression, and work pressure) (50). Both surveys in 2013 and 2018 included questions on chronic diseases, 2-week sickness, hospitalization, and commercial insurance, and we calculated the prevalence of these indicators (Table 3). From the objective aspects affecting SRH, the total prevalence of chronic diseases, 2-week sickness, and hospitalization rate in 2018 were higher than those in 2013. A related study has shown that two-week sickness, chronic diseases, and hospitalization were the most powerful predictors of SRH (51). From the subjective aspects, the total prevalence of commercial insurance in 2018 was higher than that in 2013. Insurance awareness is an important factor influencing the demand for commercial insurance (52). People with strong insurance awareness often have stronger health awareness and expectations of health or health standards. Those with low self-rated scores may have higher health expectations or higher health standards. The decreased proportion of SRHLE on LE and expansion prevalence of chronic disease, 2-week sickness, and hospitalization between 2013 and 2018 suggest that the residents of Jiangxi are experiencing “the failures of success” (9).

**Table 3:** The prevalence of SRH influencing factors in 2013 and 2018, *n* (%).

**Variable**	**2013**	**2018**	**$\chi_{CMH^{a}}^{2}$**	***P***
	**(*N* = 8,797)**	**(*N* = 7,916)**		
Chronic disease	2,131 (24.22)	2,748 (34.71)	93.27	<0.001
Two-week sickness	1,738 (19.76)	2,567 (32.43)	215.97	<0.001
Hospitalization	845 (9.61)	1,059 (13.38)	35.40	<0.001
Commercial insurance	473 (5.38)	634 (8.01)	80.28	<0.001

^a^*Cochran–Mantel–Haenszel statistics, age as a stratification factor*.

Urban–rural differences exist between urban and rural residents in Jiangxi regarding SRHLE. Urban residents have a longer LE and SRHLE than rural residents, and the absolute differences in SRHLE were higher than those in LE between urban and rural residents. From the aspect of the healthy life ratio, urban residents had a higher quality of life than rural residents; in other words, there were health inequalities between urban and rural residents. A study in Beijing, China, also confirms the existence of health inequalities between urban and rural areas (53). Urban residents have more advantages in health than rural residents. Strong mortality and functional health benefits appear in those in urban areas, some of which are explained by better access to services for individuals living in urban areas and higher socioeconomic status among urbanites (53). The National Research Council's Panel on Urban Population Dynamics (54) summarizes the health advantages of urban residents in two parts. Part of the advantage is thought to be a function of environmental factors, such as a greater concentration of health facilities, and another part is thought to be a function of individual factors, such as higher levels of income and education. Rural income has consistently lagged behind urban income, and the gap between the rich and the poor continues to widen (55). From the data of these two surveys, we found that the same situation exists in Jiangxi. In 2018, the gap in annual household income between urban and rural areas was double that in 2013, from RMB 9,734 yuan to 19,348 yuan. Looking at the urban–rural differences over time, the differences in LE decreased, but the differences in SRHLE increased from 2013 to 2018. This indicates that mortality inequality between urban and rural areas has narrowed, while inequality in quality of life has expanded. It is worth noting that this widened difference is also reflected in the average annual income of households. Although the causes of rural–urban disparities are beyond the scope of this article, our research undoubtedly provides direct evidence for rural–urban health inequalities.

Our research supports the female–male health–survival paradox (23). Women had longer LE and SRHLE than men in both 2013 and 2018, while their quality of life was lower than that of men. This is consistent with studies in Japan (8), Thailand (20), and other countries (21, 22, 26). For women with higher LE, a recent study concluded that LE for men remained lower than that for women, mainly because of the high prevalence of preventable diseases and premature deaths among men (56). Smoking is believed to be the single largest factor in explaining the gender differences in mortality in high-income countries (57). Women's mortality advantage contributes to more SRHLE in women. The most common explanations for the “gender paradox” are based on the biological and sociocultural factors that shape gender roles and the resulting healthy behavior and use of health services (58). In terms of SRH, a study in South Korea suggested that low education level was an explanatory factor for low SRH rates (19). From the data of these two surveys, the proportion of men receiving higher education in Jiangxi was higher than that of women in both 2013 and 2018. Women held more negative attitudes and reported more problems in health self-assessment than men, were more willing to admit their health problems than men, and tended to report that they were more serious (59). It is worth mentioning that there was no significant difference in the prevalence of SRH between men and women in 2013, but in 2018, there was such a difference. Seeing the gender difference from the time trend, the absolute difference in LE between men and women in 2018 was approximately the same as in 2013, but the absolute difference in SRHLE narrowed. This means that women's advantage in SRHLE has been reduced, and the quality of life of women was further reduced relative to that of men.

There are several limitations of this study. First, our study employs cross-sectional data, using age-specific health prevalence rates rather than incidence rates to calculate healthy expectancy. The use of prevalence rates will bias the estimation of healthy expectancy (60). Second, since there are no reliable age-specific mortality data, the life table of this study used the method of the China model life table and uses parameters (IMR and U5MR) for estimation, which has a certain impact on the accuracy of the output results. Third, there is no uniform SRH standard or method worldwide, so it is difficult to compare countries and regions. Different methods of SRH may lead to some differences in the results of the survey among countries and regions. Fourth, in the results of the SRH rate by gender in both urban and rural areas in 2013 and 2018, older age groups such as rural men aged 85 in 2013 and urban men aged 85 in 2018 showed sudden drops. In our original data, there were only 11 rural males aged 85+ in 2013. The sudden drop in SRH in this age group may be related to the small sample size, but it cannot be ruled out that this may be true. In 2018, there were 50 urban men aged 85 and above, and there was also a sudden drop in the SRH rate. To study this phenomenon more deeply, we need a larger sample size. This is one of the limitations of our research. Finally, the subjective nature of SRH may cause some problems, especially when comparing responses in different periods. Differences in SRH levels in different periods (different subgroups) may reflect actual differences in health conditions, but it may also be the result of differences in reports or changes in health expectations (8). In addition, people's health concepts may also change although their actual health status has not changed. Different health concepts will also affect self-evaluation of health. For instance, under the same health condition, poor individuals may feel that having no illness means that one is healthy, while the rich have a more precise definition of health.

## Conclusion

This article first calculated the SRHLE by urban–rural and gender differences of residents in Jiangxi Province of China in 2013 and 2018 and provided the first estimates of trends in SRHLE. Based on the two regionally representative surveys of health services in Jiangxi, the SRH status of residents of Jiangxi has declined, which is reflected by the decreased SRHLE and its proportion relative to LE. Our study supports the theory of expansion of morbidity. The SRHLE of women was higher than that of men; however, women's quality of life was lower, and their advantage in SRHLE has weakened. Urban residents have a higher level of health than rural residents, and this inequality has increased. Health promotion strategies should be adopted to strengthen the prevention and treatment of chronic diseases and advocate for an active and healthy lifestyle. Policy efforts are necessary to control the increased morbidity of chronic diseases and reduce gender and urban–rural differences in the quantity and quality of years lived.

## Data Availability Statement

The raw data supporting the conclusions of this article will be made available by the authors, without undue reservation.

## Author Contributions

ZL and SH: conceptualization and methodology. ZL: writing—original draft preparation and writing—review and editing. SH: supervision and project administration. ZL and HZ: formal analysis and visualization. YW and YL: software. SW: data curation. All authors have read and agreed to the published version of the manuscript.

## Conflict of Interest

The authors declare that the research was conducted in the absence of any commercial or financial relationships that could be construed as a potential conflict of interest.
