# Supplementary material for: Self-Rated Healthy Life Expectancy Changes in Jiangxi Province of China by Gender and Urban–Rural Differences, 2013–2018
Source: Front Public Health. 2021 Jan 25;8:596249. doi: 10.3389/fpubh.2020.596249 (PMC7868547; doi:10.3389/fpubh.2020.596249)
Supplement: Supplementary file 1 [file Presentation_1.pdf]

APPENDIX

Year differences in SRHLE under different SRH standards

Figure A1 shows the trends in absolute differences in SRHLE between 2013 and 2018 of people aged 15 and older under the SRH standards of ≥60, ≥70, ≥80, and ≥90. The SRHLE of both genders for almost all age groups (except for females aged 75 under the SRH standard of ≥90 and males aged 85 under the standard of ≥80 and ≥90) in 2013 was higher than that in 2018 under the SRH standard of ≥70, ≥80, and ≥90.

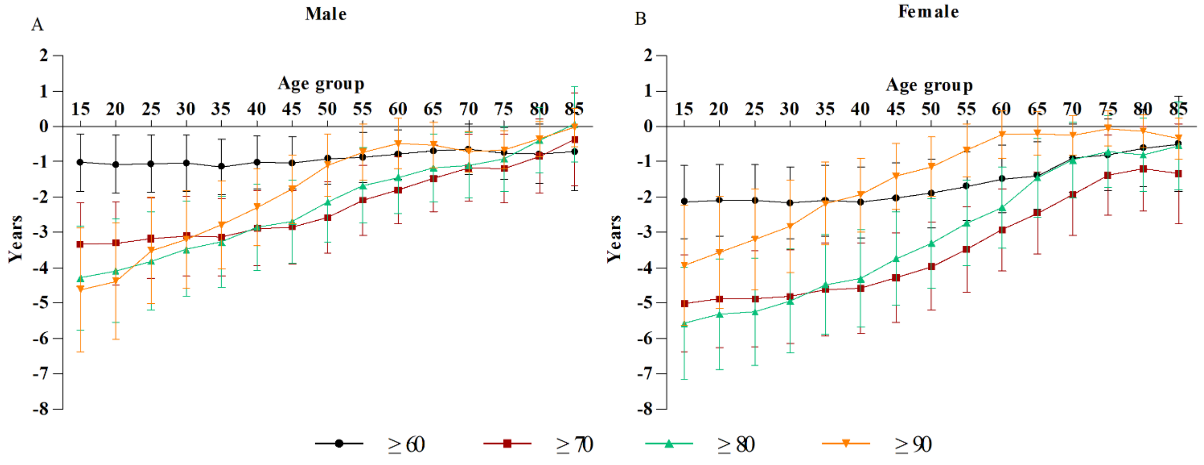

Figure A1. Trends in year absolute differences (2018 to 2013) of SRHLE and 95% CI. (A) Trends in year absolute differences of SRHLE for males. (B) Trends in year absolute differences of SRHLE for females.

Urban–rural differences in SRHLE under different SRH standards

Figure A2 shows the trends in urban–rural absolute differences in SRHLE under different SRH standards. The SRHLE of both genders in urban areas for almost all age groups (except for males aged 80+ in 2013 under the SRH standard of ≥80 and females aged 80 in 2018 under the standard of ≥90) was higher than that of rural areas in 2013 and 2018 under the SRH standards of ≥70, ≥80, and ≥90.

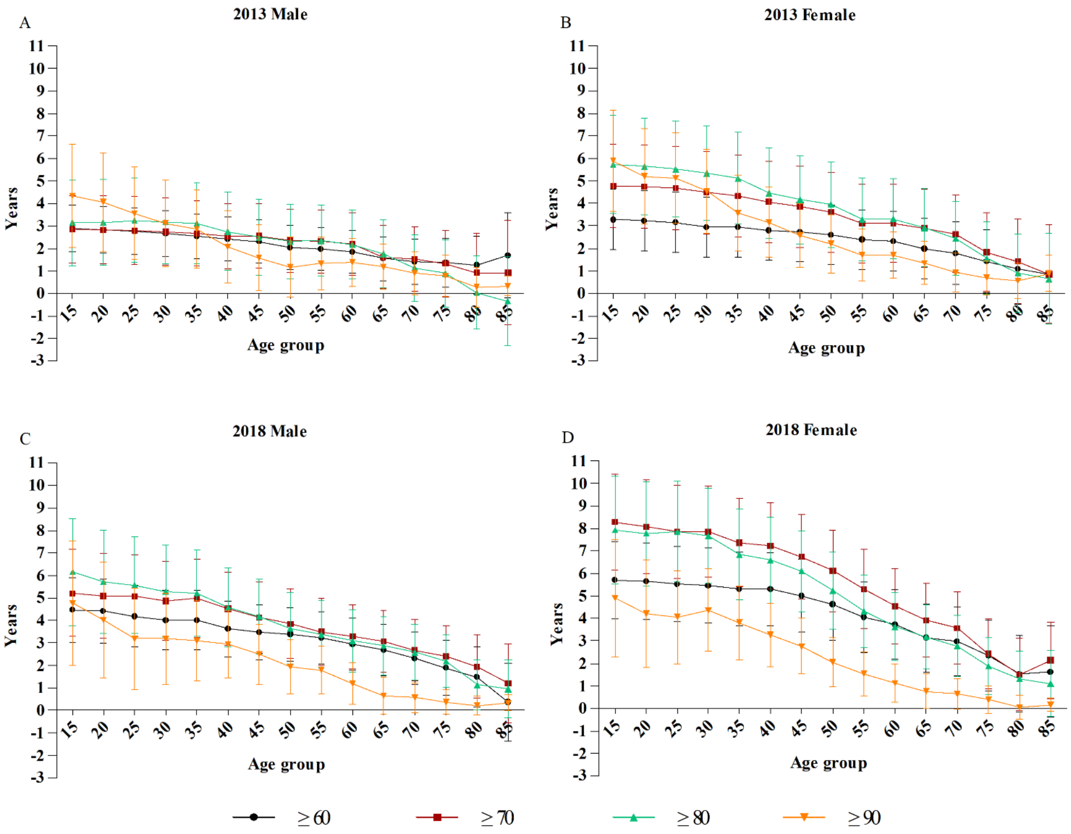

Figure A2. Trends in urban–rural absolute differences (urban to rural) of SRHLE and 95% CI. (A) Trends in urban–rural absolute differences in SRHLE for males in 2013. (B) Trends in urban–rural absolute differences in SRHLE for females in 2013. (C) Trends in urban–rural absolute differences in SRHLE for males in 2018. (D) Trends in urban–rural absolute differences in SRHLE for females in 2018.

Gender differences in SRHLE under different SRH standards

The gender differences under the SRH standards of ≥70, ≥80, and ≥90 were slightly different from the SRH standard of ≥60. Figure A3 shows the trends in absolute gender differences in SRHLE under different SRH standards. The SRHLE of females for all age groups (except aged 75) was higher than that of males in 2013 under the SRH standard of ≥70, while only females aged 15 and 20 were higher than males in 2018. When the SRH standard was increased to ≥90, the SRHLE of females (except aged 85 in 2013 and 75–84 in 2018) was lower than that of males.

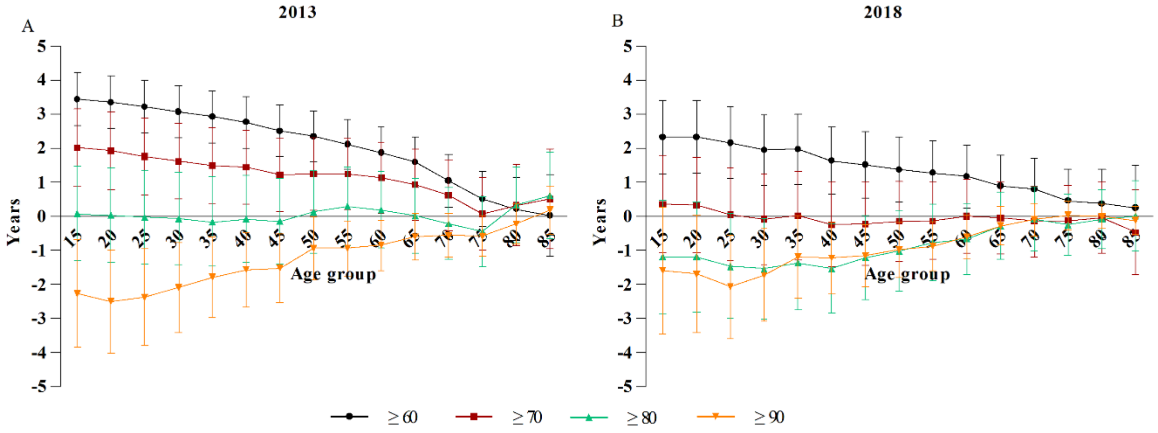

Figure A3. Trends in absolute gender differences (female to male) in SRHLE and 95% CI. (A) Trends in absolute gender differences in SRHLE in 2013. (B) Trends in absolute gender differences in SRHLE in 2018.
